# Endoscopic Management of Ampullary Adenomas: A Comprehensive Review

**DOI:** 10.3390/jcm14103532

**Published:** 2025-05-18

**Authors:** Minh Thu T. Nguyen, Ruchir Paladiya, Dushyant Singh Dahiya, Murali Dharan

**Affiliations:** 1 Division of Gastroenterology and Hepatology, Department of Internal Medicine, University of Connecticut Health Center, Farmington, CT 06030, USA; 2 Department of Internal Medicine, University of Connecticut Health Center, Farmington, CT 06030, USA; 3 Division of Gastroenterology, Hepatology and Motility, The University of Kansas School of Medicine, Kansas City, KS 66160, USA

**Keywords:** ampullary adenoma, ampullary adenocarcinoma, endoscopic ampullectomy, endoscopic retrograde cholangiopancreatography, endoscopic ultrasound

## Abstract

Ampullary adenomas are rare outgrowths at the ampulla of Vater that may progress into cancer via the adenoma-to-carcinoma sequence, particularly in individuals with hereditary polyposis syndrome. Many are diagnosed incidentally or once the lesion becomes large enough to cause obstruction. Traditionally managed surgically with high morbidity and mortality, advances in imaging and therapy have made endoscopic ampullectomy the first-line treatment for noninvasive lesions. Despite its high success rate and favorable safety profile, complications such as pancreatitis, ductal stenosis, bleeding, recurrence, and perforation can occur. Recommendations for optimal endoscopic techniques and surveillance intervals are largely based on expert opinion in interventional endoscopy and findings from small-scale studies. This review provides an updated framework for the diagnosis and management of ampullary adenomas.

## 1. Introduction

Ampullary adenomas are glandular, dysplastic polyps that arise in the ampullary complex of the duodenum, just distal to the confluence of the common bile duct (CBD) and pancreatic duct (PD). They may originate from the pancreas, CBD, duodenum, or structures of the ampulla of Vater itself, including the sphincter of Oddi and papilla of Vater. The risk of malignancy increases with lesions larger than 10 mm, with up to half potentially harboring small foci of adenocarcinoma [1]. The malignant potential of these neoplasms, combined with their remote location, makes their identification and staging challenging.

Over the past decade, the treatment for ampullary adenomas has shifted from surgery to endoscopic ampullectomy (EA). However, variations in diagnosis, staging, resection, and surveillance persist due to the rarity of the pathology and lack of large studies. Complications from EA are more significant than those seen with other endoscopic procedures, highlighting the need for expertise. This review aims to provide an updated overview of diagnosis and management of ampullary adenomas.

## 2. Ampullary Adenomas

### 2.1. Epidemiology

**Figure 1:**
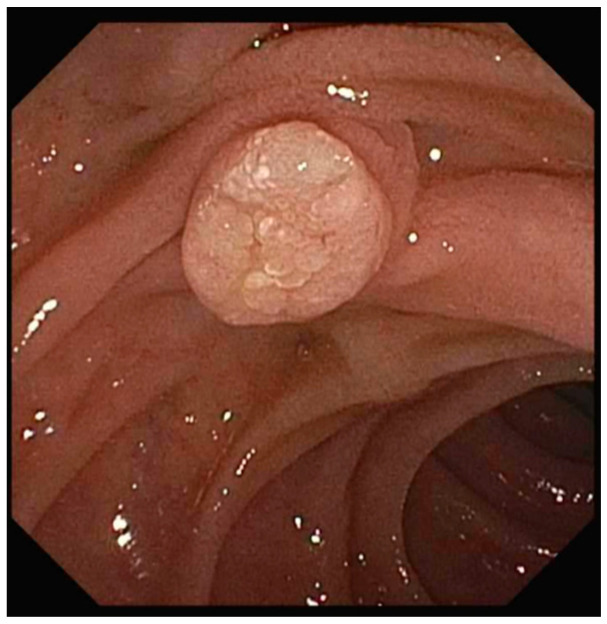
Example of an ampullary lesion.

**Figure 2:**
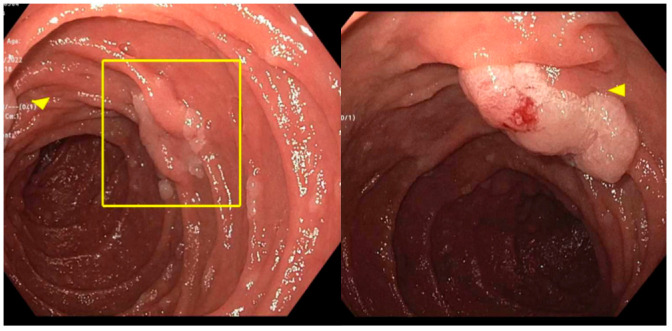
Example of an ampullary adenoma (yellow box) in a patient with familial adenomatous polyposis (FAP), accompanied by multiple other duodenal adenomas (yellow arrows).

Neoplasms are more common in the ampullary region than in any other area of the small intestine (Figure 1) [2]. Ampullary adenomas have an estimated incidence of 0.04–0.12%, based on post-mortem autopsy series [3]. Sporadic ampullary adenomas are most commonly detected in patients over 40 years old, with peak prevalence occurring in the sixth to seventh decades of life [4]. In cases of genetic predisposition observed in familial adenomatous polyposis (FAP), hereditary nonpolyposis colorectal cancer (HNPCC), and mutY DNA glycosylase (MUTYH)-associated polyposis, they are often identified earlier because of heightened screening recommendations (Figure 2) [4]. Although adenomas account for most ampullary masses, a range of non-neoplastic, benign, and malignant lesions must also be considered (Table 1) [5].

### 2.2. Premalignant Potential

**Figure 3:**
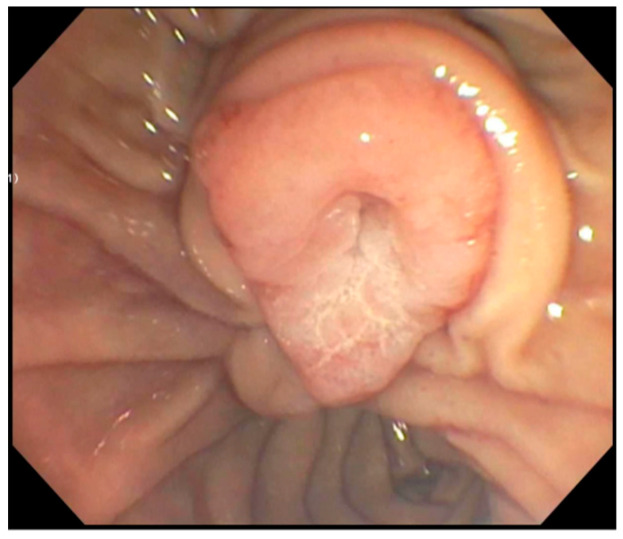
Example of a large ampullary adenoma with predominant intraductal extension.

**Figure 4:**
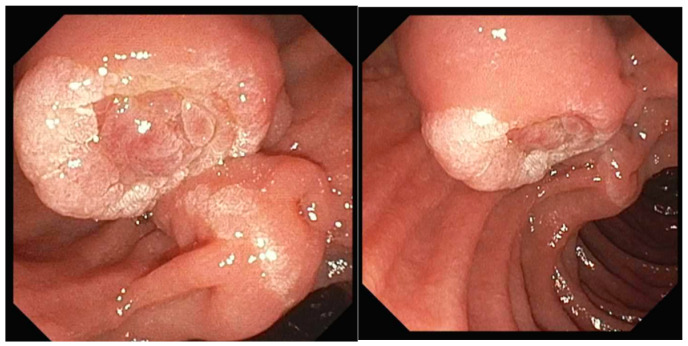
An ampullary adenoma with intraductal extension was identified. Following surgical intervention, the final pathology revealed an ampullary adenocarcinoma staged as pT2 N0 (Stage 1B).

**Figure 5:**
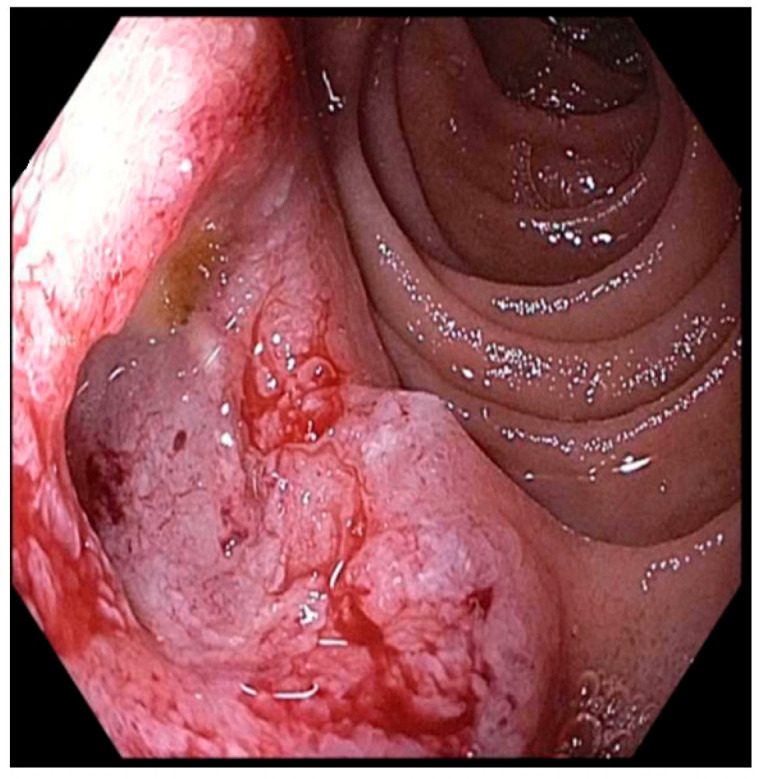
Example of an ampullary adenocarcinoma presenting as a large, irregular, friable mass prone to bleeding.

Ampullary adenomas are premalignant lesions, with cancer foci present in 15–60% of cases (Figure 3) [6]. They can progress to ampullary adenocarcinomas, which make up about 0.5% of gastrointestinal neoplasms (Figure 4 and Figure 5) [4]. Ampullary adenocarcinoma most commonly arises in an analogous fashion to colorectal cancer, following a well-documented adenoma–carcinoma sequence [2]. Patients with FAP have an autosomal dominant mutation in the adenomatous polyposis coli (APC) gene that predisposes them to developing numerous adenomas [7]. This population has a lifetime risk of developing duodenal adenomas approaching 90%, with a cumulative possibility of adenocarcinoma up to 10% [7]. Since many undergo early proctocolectomy for cancer prevention, ampullary adenocarcinoma is the leading cause of death in post-colectomy FAP patients [7].

### 2.3. Clinical Manifestations

Ampullary adenomas are typically discovered incidentally during endoscopy or imaging, as most patients are asymptomatic [2]. Symptomatic cases usually result from mass effect due to compression of the nearby biliary and pancreatic ducts [2]. Common clinical manifestations include jaundice and epigastric pain. Less typical symptoms include nausea, vomiting, weight loss, early satiety, abdominal distension, biliary colic, pruritis, or cholangitis [8]. Rarely, patients may present with acute pancreatitis, iron deficiency anemia, or upper gastrointestinal bleeding [8].

## 3. Endoscopic Ampullectomy (EA)

### 3.1. Surgery

Ampullary adenomas were traditionally treated with the Whipple procedure (pancreaticoduodenectomy), which involves removal of the pancreatic head, duodenum, jejunum, gallbladder, and gastric antrum [9]. Given its extensive nature, the Whipple procedure is associated with high morbidity and mortality (50–65% and 0–9%, respectively) [10,11]. The benefit of radical resection is that it removes local lymph node metastases, which may remain undetected, and eliminates the need for endoscopic surveillance in patients without adenomatous polyposis syndrome [9]. It is curative for patients with sporadic adenomas or non-metastatic periampullary cancers [9].

Transduodenal surgical ampullectomy (TSA) is a minimally invasive surgery [9,11]. Its morbidity and mortality rates are lower than those of pancreaticoduodenectomy but remain significant (14–27% and 0–4%, respectively) [10,11]. Recurrence rates are high (5–30%), warranting continued endoscopic surveillance [3,9,10].

A study of 30 patients found that EA was associated with shorter intervention times, less blood loss, fewer complications, lower morbidity, and shorter hospital stays compared to surgery. However, re-intervention rates were higher in the endoscopic group [12]. A meta-analysis reported a complete resection rate of 76.6% (R0) and a recurrence rate of 13.0% for EA, compared to a 96.4% R0 rate and a 9.4% recurrence rate for TSA [13]. Another multicenter study found higher R0 resection rates in the TSA group (90.5% vs. 73.1%; *p* < 0.001) than in the EA group, even after additional ablation in the latter [14]. The study also reported a higher recurrence rate (16%) in the EA group than in the TSA group [14].

Despite these limitations, EA is recommended for benign lesions due to its favorable safety profile and shorter recovery time [14]. Surgery, while associated with higher morbidity (28.3%) and longer recovery, is considered when EA is not feasible or has failed [13]. A pooled analysis of three datasets showed that pancreaticoduodenectomy had the highest R0 resection rate (98.9%) and recurrence rate (14.2%) but also the highest morbidity (44.7%) and mortality [13]. It is typically reserved for high-risk or malignant lesions because of its extensive nature and high complication rate [14]. However, existing studies are heterogeneous and difficult to compare, which leaves room for informed discussion between the clinician and the patient.

### 3.2. Emergence of EA

EA was first described in 1983 and later detailed in a case series by Binmoeller et al. in 1993 [15]. Since then, EA has become the first-line therapy for excision of ampullary adenomas [16]. It requires advanced endoscopy training, limiting its availability in academic and tertiary referral centers. “Endoscopic ampullectomy” and “endoscopic papillectomy” are often used interchangeably, although the latter is technically more precise [16].

## 4. Pre-Operative Imaging

Abdominal ultrasound (US) is the first choice for evaluating obstructive jaundice due to its speed, cost, and availability. However, it often misses ampullary adenomas, relying on secondary signs such as duct dilation, and is limited by gas interference, making it inadequate for staging [17]. Contrast-enhanced abdominal computed tomography (CT) can detect soft tissue masses, including those at the ampulla, irregular margins, and dilated ducts [18]. However, CT has low sensitivity for smaller neoplasms, with a reported accuracy of only 20% [17]. Although not sensitive enough for local staging, helical CT is useful for detecting regional lymphadenopathy and distant metastases [19].

Magnetic resonance imaging (MRI) and magnetic resonance computed tomography (MRCP) are often used as noninvasive methods to investigate ductal abnormalities observed on US or CT [18]. They can distinguish ampullary adenomas as filling defects or a dilated CBD with distal focal stricture [18]. However, these imaging methods are non-diagnostic, as they cannot directly visualize the ampulla or allow for tissue sampling. Although liver function tests may indicate cholestatic injury from obstruction, no specific laboratory test or tumor marker for ampullary adenomas currently exists.

## 5. Diagnostic Testing

Diagnosis is confirmed through histopathology using a high-definition side-viewing endoscope and should be established before resection [20]. Direct macroscopic inspection alone is insufficient, as adenomas and adenocarcinomas may visually resemble other ampullary lesions [21]. Benign polyps typically have regular margins, soft consistency, and no ulcerations or bleeding [4,22]. Features of malignancy include induration, rigidity of the papilla upon probing, mucosal ulceration, and erythema [23]. In one study of 56 patients, failure to achieve an uplifted cleavage plane with submucosal injection was the strongest predictor of malignancy [24].

Several forceps biopsies should be obtained to maximize diagnostic accuracy [4]. If the lesion contains multiple folds, orienting the forceps parallel to the folds can decrease reactive fibrosis [25]. Random ampullary biopsies are also recommended, as normal-appearing papillae may harbor precancerous changes. To avoid inducing pancreatitis, tissue should ideally be sampled at the 10–12 o’clock position, away from the pancreatic orifice [4,26]. Biopsies can also be obtained from pancreatic and biliary intraductal segments. If the patient is undergoing concomitant endoscopic retrograde cholangiopancreatography (ERCP), obtaining biopsies after sphincterotomy improves diagnostic yield [16]. Brush cytology and fine-needle aspiration (FNA) may be used alongside forceps biopsies, but there are no data to support their standalone use in this setting [27]. A consensus guideline reported diagnostic rates (adenoma or adenocarcinoma) of 45–80% among ampullary biopsies, with false-negative results seen in 16–60% of patients with adenocarcinoma [3,28,29]. Complete resection definitively rules out foci of malignancy; one study reported a 64% diagnostic agreement between pre-ampullectomy biopsy and ampullectomy pathology [30].

Some endoscopists apply chromoendoscopy with non-absorptive dyes (most commonly indigo carmine) to help determine the margins of flat lesions, although this is not routine practice [16,20]. When available, optical enhancements with white light, magnification, and narrow-band imaging (NBI) allow for direct visualization of underlying irregular microvessels and mucosal microstructures, further complementing diagnostic precision [31,32]. Biopsy samples may be subjected to immunohistochemical staining for the p53 tumor suppressor gene, polymerase chain reaction (PCR) for the *K-ras* gene mutation, microRNA expression analysis, and flow cytometry to enhance diagnostic accuracy [16,33,34]. It is important to have an experienced pathologist to review the specimen, as intra-observer variability is common [19]. Patients with biopsies positive for adenocarcinoma should be promptly referred to an oncologic or hepatobiliary surgeon.

## 6. Staging

Small, non-malignant lesions less than 20 mm in size can generally be resected without extensive evaluation. However, comprehensive multimodal staging is crucial before endoscopic removal. Various imaging modalities, such as endoscopic ultrasound (EUS), MRCP, and ERCP, can be used to define the type and extent of the lesion. These modalities are particularly effective for evaluating submucosal invasion, lymph node metastasis, and the extent of intraductal invasion [35].

### 6.1. Endoscopic Retrograde Cholangiopancreatography (ERCP)

ERCP is important for local staging and can be performed immediately before EA [36]. It allows for visualization of any intraductal spread of the ampullary lesion into the pancreatic and biliary ducts using fluoroscopy [36]. ERCP may also serve as a therapeutic option for biliary decompression following stent placement if there is evidence of obstruction.

### 6.2. Endoscopic Ultrasound (EUS)

EUS is reserved for evaluating large ampullary adenomas (≥2 cm) or lesions with malignant features on endoscopy [26,37]. It is used for preoperative staging of ampullary cancers [37]. EUS has been shown to be superior to CT, MRI, and US for this purpose, although not significantly superior to IDUS [38]. In one study, the sensitivity and specificity of EUS for detecting intraductal extension were 80% and 93%, respectively, comparable to 83% and 93% for ERCP [39]. Using a linear echoendoscope, EUS can also assess lesion depth and local lymph node involvement [40].

### 6.3. Intraductal Ultrasound (IDUS)

Endoscopy alone faces many challenges in precise staging, including difficulty distinguishing adenomatous tissue from normal papillary tissue, optical distortions that skew diameter measurements, and partial visual occlusion of adenomas by the rim of the papilla [41]. IDUS is useful for the detailed evaluation of narrow ductal cavities and neighboring structures because of its higher-frequency waves (12–30 MHz), which produce high-resolution images within a 2 cm radius [42]. IDUS is performed using a small, thin-caliber probe that can be inserted during cannulation in ERCP, usually after sphincterotomy [42]. This modality is superior to ERCP alone for staging ampullary adenomas [26,43]. The diagnostic accuracy of T staging in ampullary cancers ranges from 62–90% for EUS and 78–93% for IDUS [41,43]. However, IDUS is not available at many institutions, increases the risk of pancreatitis, and may overestimate tumor staging [43]. It is recommended only when there is strong clinical suspicion of tumor extension, as its use could guide the decision between surgical and endoscopic management.

## 7. EA Techniques

### 7.1. Indications

EA techniques depend on lesion size, intraductal involvement, spread, polyposis syndrome, and endoscopist’s preference. Risks and benefits should be discussed with patients, considering age, comorbidities, life expectancy, and follow-up ability. For those not opting for intervention, close observation with imaging, alone or combined with endoscopy, is a reasonable approach. Alternatively, patients who are too ill or have a limited prognosis for any reason may be best served by foregoing endoscopic ampullectomy or surgery altogether.

Ampullary adenomas can generally be removed endoscopically if they are ≤3 cm in size, have ≤1 cm extension into the bile or pancreatic duct, possess benign histology, and show no signs of advanced duodenal polyposis [26]. Exceptions may apply for experienced endoscopists, with some ampullary adenomas >3 cm safely resected [44]. While endoscopic resection of well-differentiated ampullary adenocarcinoma is increasingly performed, it should be performed only by experts due to the risk of residual neoplasia in surrounding structures and lymph nodes [45,46]. Lesions with moderately to poorly differentiated adenocarcinoma should not be treated with EA and should instead be referred for surgery [1].

### 7.2. Procedure Set-Up

Similar to other endoscopic procedures, patients must fast overnight and usually require moderate-to-deep sedation. General anesthesia may be needed if prolonged procedure time is anticipated for complex lesions. Prior clearance from cardiology or pulmonology is recommended for patients with cardiopulmonary comorbidities.

A standard therapeutic duodenoscope can be used in most cases, though some experts prefer a larger one to allow easier passage of snare and thermal probes, as well as for aspirating air and argon gas [16]. Features such as NBI and high-definition optics enhance lesion inspection.

At the start of the procedure, the lesion is first inspected for signs of malignancy and probed for firmness and mobility. The pancreatic and bile ducts are cannulated and partially filled with contrast to assess ductal extension and anatomy, and to prepare for possible stent placement. Optionally, diluted methylene blue is injected to outline the pancreatic duct for easier post-resection identification [47].

### 7.3. Endoscopic Resection

Lesions located solely in the papilla with clear margins should be resected en bloc to ensure complete resection and preserve the specimen integrity for histopathology [48]. This also reduces procedural time, minimizes cauterization, and decreases reactive fibrosis [48]. Both standard braided polypectomy snares and fine-wire snares can be used, though thin, rigid snare wires may offer better resection depth, especially when incising the sphincter of Oddi [49]. Snare positioning can be cephalad-to-caudal or caudal-to-cephalad. The snare tip is anchored above the papilla before snaring the entire mass. Once the mobility is confirmed, the snare is firmly closed for en bloc resection.

Complete resection is deemed successful if no residual lesion is visible or confirmed histologically during initial follow-up. Lesions larger than 2 cm, with irregular shapes or lateral spread, may require piecemeal resection with endoscopic mucosal resection (EMR) or endoscopic submucosal dissection (ESD), often necessitating multiple attempts [50].

If a lateral spread is present, it is first removed to isolate the ampullary lesion [50]. Case reports have described cold snare polypectomy (CSP) for resecting lateral spreading ampullary growths, as opposed to hot snare [50,51]. This approach can potentially reduce the incidence of thermal injury complications, delayed hemorrhage, and perforation risk [51,52]. However, CSP of a laterally spreading component may carry a risk of residual disease [53].

### 7.4. Submucosal Injection

Submucosal injection separates the targeted lesion from the underlying muscular layer, allowing for easier removal, reduced thermal injury, and a decreased risk of bleeding or perforation [54]. This technique is commonly used for large or laterally spreading growths, typically during EMR or ESD [54]. However, there is no consensus on the routine use of submucosal injections in EA. Similarly, the injection volume and type of solution are not standardized.

A prospective multicenter study by Hyun et al. found that injection with 1:10,000 diluted epinephrine prior to EA offered no advantage in terms of achieving complete resection or reducing the incidence of adverse events such as bleeding [55]. Administering epinephrine via injection at the inferior (caudal) aspect of the ampullary lesions can clearly delineate the inferior margin. Elevating the caudal component may facilitate the “fulcrum technique”, in which the tip of an open snare, embedded superficially in the duodenal mucosa at the cranial aspect of the lesion, engages the entire ampullary lesion flush with the duodenal wall [56,57].

Submucosal injections for lifting are particularly challenging and problematic at the ampulla due to its complex anatomy. The duodenal papilla contains the ampulla of Vater, which is a confluence of the biliary and pancreatic ducts that terminates in the muscularis propria of the duodenal wall [58,59]. Ampullary adenomas are tethered to the ducts and may develop ballooning and mushroom effects with injection, which can misalign the snare and hinder complete excision [58]. There are reports of increased rates of post-resection pancreatitis with injections [10]. We do not recommend submucosal injection unless there is evidence of extra-ampullary extension or lateral spread beyond the ductal openings. When necessary, we prefer to restrict lifting injections to the areas surrounding the ampulla, avoiding direct injection into the center.

### 7.5. Electrocautery Settings

The optimal electrocautery settings have not been firmly established. Before the widespread availability of the blended current ENDOCUT^®^ (Erbe), pure cutting current AUTOCUT^®^ (Erbe) was preferred, as it enabled swift lesion removal with less collateral damage to the ducts. Now preferred at many institutions, the ENDOCUT^®^ setting combines cutting and coagulation currents in short, alternating bursts with pauses, automatically adjusting output to overcome tissue resistance [60]. Exact parameters may vary depending on the specific equipment; however, a commonly used preset includes a pure cut current with an output limit of 120 W and a soft coagulation current with an output limit of 30 W [60]. One study recommends using an ENDOCUT^®^ setting of effect 1, duration 4, and interval 1 [49].

A prospective, single-blind trial by Iwasaki et al. demonstrated that both pure cutting and blended current modes have similar efficacy and safety; however, the blended current reduces the risk of immediate bleeding in patients with large lesions (≥14 mm diameter) [61]. Conversely, the ENDOCUT^®^ mode may increase the risk of postoperative pancreatitis by causing thermal tissue damage, as well as blurring tumor margins and hindering the assessment of vertical invasion depth [60].

### 7.6. Retrieval of Resected Specimen

The resected specimen should be promptly retrieved using a snare or Roth net to prevent it from migrating distally into the jejunum due to peristalsis [10]. Air insufflation and aspiration should be avoided to prevent specimen fragmentation and potential loss of malignant foci. Antispasmodics such as glucagon and scopolamine-butyl may help prevent specimen loss. Positioning the patient prone encourages the specimen to move toward the duodenal bulb due to gravity.

### 7.7. Ablative Therapies

After specimen collection, the duodenoscope is reinserted at the papillary site to carefully inspect for bleeding or residual polyp tissue. While ablation is not routinely recommended, it may be used in cases of evident residual tissue or recurrence. Argon plasma coagulation (APC) is the preferred method, though other options include laser therapy, photodynamic therapy, and monopolar/bipolar electrocoagulation.

A disadvantage of ablation is that it can destroy lesion margins, making it technically difficult to obtain clean biopsy specimens. To date, studies have not shown a significant benefit from adjunctive therapy. In one study involving 103 patients, the success rates of EA with (81%) and without (78%) thermal ablation were comparable [48]. Furthermore, thermal therapy is not recommended as a standalone method for resection.

Radiofrequency ablation (RFA) has been studied for recurrent ampullary adenomas, especially in cases with residual neoplasia after EA. A multicenter study by Camus et al. evaluated intraductal RFA in 20 patients with confirmed endobiliary adenoma remnants, showing a 70% dysplasia eradication rate at 12 months with a good safety profile [62]. Adverse events occurred in 40% of patients, but none were major [62]. Rustagi et al. found a 92% treatment success rate in 14 patients with ampullary neoplasms and intraductal extension, with 100% success in those treated solely with RFA; ductal strictures were common but manageable with stent therapy [63].

### 7.8. Pancreatic and Biliary Stenting

Experts recommend PD stenting shortly after excision as a prophylaxis against post-ampullectomy pancreatitis. Prophylactic stenting is considered anecdotally effective and is based on small-scale studies. Pancreatic stenting may also reduce the risk of reactive stenosis in the PD orifice. We always perform PD stenting unless contraindicated, usually with a 5 French, 3 cm plastic stent. As an additional measure, we administer a one-time dose of rectal indomethacin suppository (100 mg). Rectal indomethacin is well-proven to significantly reduce the incidence of post-ERCP pancreatitis. The PD stent is then retrieved 1–2 weeks later via EGD using rat-tooth grasping forceps, if it has not fallen out already (usually seen on abdominal X-ray). We recommend routine placement of PD stents in patients without pancreatic divisum.

Since postprocedural cholangitis is rare, prophylactic biliary stenting is recommended only on a case-by-case basis—typically when there are concerns about inadequate biliary drainage or the presence of biliary stones or sludge. For instance, biliary stenting may be beneficial in the event of major bleeding to facilitate bile drainage or when there is concern for microperforation. These plastic stents are usually removed after 2–3 weeks. During EA, resection of any biliary intraductal ingrowth should generally be avoided to minimize excessive manipulation and reduce the risk of shearing the resection site, which could lead to immediate hemorrhage.

### 7.9. Pancreatic and Biliary Sphincterotomy

Routine sphincterotomy of either duct is not recommended. When intraductal extension of the lesion is visualized, a small sphincterotomy may be performed to expose the distal CBD. A balloon-tipped catheter can then be used to evert the intraductal segment outward into the CBD, facilitating resection. Aggressive sphincterotomy may cause tissue injury to the pancreatic and biliary orifices, increasing the risk of ductal stenosis, bleeding, or infection.

Some centers prefer performing a biliary sphincterotomy before EA, as it allows the PD guidewire to remain in place and facilitates passage of the snare over the wire for polyp resection [64]. Biliary sphincterotomy also helps prevent the specimen from dislodging before placement of the PD stent, enabling immediate retrieval. Additionally, biliary sphincterotomy may mitigate the risk of obstruction caused by reactive edema or clots. Maintaining a PD guidewire after resection ensures continued ductal access and supports timely placement of a PD stent [64].

### 7.10. Post-Procedural Care

After EA, post-procedural care is influenced by lesion location, procedure duration, and the endoscopist’s experience. One group with extensive experience observed patients for 4 h before discharging them the same day [65]. More commonly, patients are admitted overnight to an observation unit, kept on nothing by mouth for 4 h, and started on maintenance intravenous fluid for hydration. An oral proton pump inhibitor (PPI), typically pantoprazole 40 mg twice daily, is prescribed for 4–8 weeks [4]. Antiplatelet agents are usually held for 7 days. The diet is advanced as tolerated, starting with clear liquid the following morning. If no complications are observed, patients may be discharged with close follow-up in the GI clinic a month later. Patients are also instructed to avoid non-steroidal anti-inflammatory drugs (NSAIDs) for one week to reduce the risk of post-procedural bleeding.

## 8. Adverse Events

Although endoscopic approaches have a safer profile compared to surgical alternatives, endoscopists must be aware of potential complications, including immediate or delayed bleeding, pancreatitis, cholangitis, stenosis, perforation, recurrence, and the need for emergency surgery.

### 8.1. Pancreatitis

Pancreatitis is a common potential complication of EA, primarily due to direct irritation of the pancreatic orifice, with an incidence ranging from 3 to 25%. It typically occurs within the first 12 h after the procedure. Special care should be taken when sampling small lesions near the pancreatic orifice (at 5 o’clock position with the papilla is viewed en face), especially in patients with FAP. Pancreatitis classically presents as moderate-to-severe epigastric pain, accompanied by at least a threefold elevation in amylase and lipase levels or radiographic evidence of pancreatic inflammation.

The mainstay treatment is supportive and includes aggressive intravenous hydration, bowel rest, pain alleviation, and nausea control. Current consensus recommends placement of a prophylactic PD stent shortly after polyp removal, along with a single 100 mg dose of rectal indomethacin. A study evaluating PD-wire-guided EA reported a 90% complete resection rate and an 8% incidence of mild pancreatitis, with no procedure-related mortality [46]. However, this technique requires technical expertise and may not be feasible in all patients [66].

### 8.2. Bleeding

Immediate bleeding occurs in 0 to 25% of cases, depending on the lesion size, techniques, and endoscopist’s experience [67,68]. It can be managed using standard hemostatic methods, such as hemoclipping and thermocoagulation. Delayed bleeding, requiring ED evaluation or hospitalization within 14 days, is treated with monitoring, bowel rest, intravenous PPIs, transfusions, and repeat endoscopy, if needed. Most patients were managed endoscopically; however, severe or refractory bleeding may require interventional radiology with embolization of the gastroduodenal artery [68].

Lateral spreading growth larger than 3 cm has a higher potential of delayed gastrointestinal bleeding, with reported rates of up to 30% [52,68]. Recent studies have also supported the use of hemostatic sprays for mucosal defects associated with large lateral spreading growths [52].

A multicenter randomized controlled trial reported a decreased incidence of delayed bleeding in the prophylactic clipping group compared to the no-clipping group (15.0% vs. 31.6%), although the results were not statistically significant [69]. Furthermore, a small prospective study demonstrated that prophylactic closure of the frenulum following EA reduced delayed bleeding without increasing procedure duration, pancreatitis risk, or perforation rates [70]. Another study recommended avoiding prophylactic hemostatic clipping unless complete mucosal closure could be achieved, as it might promote shearing of the mucosal defect and lead to immediate bleeding. Additionally, there is a potential risk of perforation due to the fixed nature of the duodenum at this location and the increased likelihood of tearing the muscularis propria fibers [52].

### 8.3. Cholangitis

Acute cholangitis following EA is uncommon (0–2%) and may be secondary to poor bile duct emptying, procedural manipulation, or stent dysfunction due to migration or obstruction [71,72,73,74]. Therefore, prophylactic biliary stenting is not routinely recommended. Cholangitis typically develops within the first few days following EA. In most cases, conservative management with intravenous hydration and a course of broad-spectrum antibiotics is sufficient to resolve the complication [75]. However, if imaging reveals biliary tract obstruction or dilation, urgent biliary decompression is required.

### 8.4. Stenosis

Stenosis of the pancreatic, biliary, or common duct can occur following procedural manipulation. Reported rates of orifice stenosis after EA range from 0–8% [74,76,77]. One study found higher odds of papillary stenosis in patients who did not undergo PD stent placement compared to those who did (15.4% vs. 1.1%) [78]. Patients who develop obstructive symptoms may require additional interventions such as sphincterotomy, stent placement, or balloon dilation via ERCP [15,75].

### 8.5. Perforation

As with all endoscopic procedures, perforation is a rare but potential complication, with reported rates ranging from 0–8% [76,79]. The thinness of the duodenal wall makes it more prone to perforation, especially at deep resection sites, which require close monitoring. Endoscopic visualization is less reliable for assessing these areas; therefore, a high index of clinical suspicion should remain high post procedure. Larger lesions and patient-related factors—such as age and comorbidities—may increase the risk of perforation, although data quantifying this risk are limited [80]. If perforation is suspected, fluoroscopy with contrast injection may be performed for evaluation; however, retroperitoneal perforations may be missed [80,81]. Persistent postoperative pain warrants radiological assessment.

The gold-standard imaging modality is CT of the abdomen and pelvis with oral contrast, due to its high sensitivity and specificity in identifying intraperitoneal or retroperitoneal gas [82]. Isolated retroperitoneal gas is more indicative of periampullary perforation, whereas intraperitoneal gas represents an uncontained leak [83,84]. Carbon dioxide insufflation may be used to reduce the risk of tension pneumoperitoneum by absorbing escaped gas [25]. As with any upper gastrointestinal perforation, the patient should be admitted, receive intravenous fluids, be kept on nothing by mouth, administered broad-spectrum antibiotics (covering Gram-negative and anaerobic organisms), and surgery should be consulted as needed.

Recent studies have reported the use of fully covered self-expanding metal stents (FCSEMS) in the CBD, instead of plastic stents, for managing microperforations in the papillary region following resection [85,86]. These stents are usually left in place for two weeks, although the optimal duration remains unclear. Standard endoscopic closure techniques, such as through-the-scope or over-the-scope clips, can also be utilized. A nasoduodenal tube may be used for larger perforations, while nasobiliary tubes are rarely used. A multidisciplinary approach, including potential surgery, is crucial for achieving optimal outcomes [35].

### 8.6. Death

The procedure-associated mortality rate following EA is very low, typically under 1% [67]. In a study by Tringali et al., a mortality rate of 0.7% was reported in a cohort of 135 patients [67].

## 9. Endoscopic Outcomes

### 9.1. Success Rate

Reported technical success rates of EA vary widely, ranging from 46–92%, and are primarily derived from retrospective and heterogeneous case series [68]. Factors predicting success include the experience of the interventional endoscopist, the techniques used, and the characteristics of the adenoma, such as size and lateral spread [68].

### 9.2. Recurrence

Recurrence of sporadic ampullary adenomas is common even after suspected complete endoscopic removal. The pooled recurrence rates for EA were 13.0% at 1 year, 12.5% at 2 years, 13.3% at 3 years, and 15.7% at 5 years [87]. According to one study, the 5-year recurrence rate was 16.9% (22 cases), with 5.9% (5 cases) requiring surgery; intraductal extension on preoperative diagnosis was the only significant risk factor for both recurrence (*p* < 0.001) and surgical intervention (*p* = 0.005) [1]. Other risk factors for recurrence include piecemeal resection and a diagnosis of FAP [1]. Therefore, periodic endoscopic follow-up and surveillance are recommended, provided they align with the patient’s care goals.

## 10. Surveillance

Patients who undergo excision of ampullary adenomas should have an endoscopic surveillance plan to assess for recurrence. For sporadic ampullary adenomas, there are no standardized surveillance intervals [16]. For patients with a large adenoma (defined as ≥2 cm) or high-grade dysplasia, we recommend duodenoscopy without ERCP within 1–3 months of excision. If there is no evidence of recurrence, surveillance may be performed every 6 months to every 2 years. For individuals with smaller adenomas (<2 cm) or low-grade dysplasia, the initial follow-up can be up to 6 months after excision. In the absence of recurrence, surveillance can be performed every 1–2 years. The interval duration should consider factors such as dysplasia grade, resection completeness, involvement, age, family history, and recurrence risk. Beyond the first two years, clinical benefits and optimal surveillance intervals remain unclear; therefore, it is important for the endoscopist to have an informed discussion with the patient about their preferences [10,16].

For patients who undergo endoscopic or surgical treatment for sporadic ampullary adenomas, we recommend surveillance with upper endoscopy every 6–12 months, including cold forceps biopsy of the papilla. Studies have shown an increased risk of colorectal neoplasia in these patients; therefore, a screening colonoscopy should be performed if not already performed [88]. For those who are too unfit for intervention, further imaging or procedures may be avoided.

We recommend using the modified Spigelman score for staging duodenal polyposis secondary to hereditary syndromes such as FAP (Table 2) [89]. The stage determines the surveillance interval (Table 3) [89]. In the case of FAP, small and asymptomatic ampullary adenomas < 1 cm in size can undergo surveillance only [7]. Individuals with FAP should begin screening for gastric and duodenal abnormalities with a duodenoscope at the age of 20–25 years or prior to colectomy [7].

## 11. Conclusions

Ampullary adenomas are rare and generally asymptomatic polyps that are increasingly detected, particularly during surveillance for polyposis syndrome. Due to their precancerous nature, careful diagnostic evaluation and preoperative staging are essential. EA is now the first-line treatment for noninvasive ampullary masses, offering a less invasive alternative to surgery. EA is a technically challenging procedure due to the complex anatomy, thin duodenal wall, and highly vascular tissue layers, and can lead to complications when performed by inexperienced endoscopists. Given the rarity of these lesions and the variability in endoscopic methods, collaborative, multicenter studies are needed. Future research should focus on improving diagnosis, staging, endoscopic techniques, and surveillance guidelines to monitor recurrence in sporadic lesions.

## Figures and Tables

**Table 1:** Differential diagnosis of ampullary lesions.

Non-Neoplastic	Benign	Malignant
Impacted gallstone Hyperplasia Hamartoma Adenomyoma Choledochocele Lymphocele Duodenal duplication Gastric heterotopia	Sporadic ampullary adenoma Ampullary adenoma associated with polyposis syndrome Lipoma Neurofibroma Granular cell tumor Lymphangioma Leiomyoma Leiomyofibroma	Ampullary adenocarcinoma Metastatic cancer Carcinoid tumor Sarcoma Leiomyosarcoma Gangliocytic paraganglioma Gastrointestinal stromal tumor (GIST)

**Table 2:** Modified Spigelman score for staging duodenal polyposis.

Factor	1 Point	2 Points	3 Points
**Number of polyps**	<4	5–20	>20
**Polyp size (mm)**	1–4	5–10	>10
**Histology**	Tubular	Tubulovillous	Villous
**Dysplasia**	Low-grade	Not applicable	High-grade

**Table 3:** Surveillance intervals based on modified Spigelman stage.

Modified Spigelman Stage	Total Points	Frequency of Surveillance
0	0	Every 4 years
I	<4	Every 2–3 years
II	5–6	Every 1–3 years
III	7–8	Every 6–12 months
IV	9–12	Every 3–6 months Consider surgical evaluation

## Data Availability

Not applicable.

## Abbreviations

CBD	common bile duct
PD	pancreatic duct
EA	endoscopic ampullectomy
FAP	familial adenomatous polyposis
HNPCC	hereditary nonpolyposis colorectal cancer
MUTYH	mutY DNA glycosylase
APC	adenomatous polyposis coli
RFA	radiofrequency ablation
TSA	transduodenal surgical ampullectomy
US	ultrasound
CT	computed tomography
MRI	magnetic resonance imaging
MRCP	magnetic resonance computed tomography
ERCP	endoscopic retrograde cholangiopancreatography
FNA	fine-needle aspiration
NBI	narrow band imaging
PCR	polymerase chain reaction
EUS	endoscopic ultrasound
IDUS	intraductal ultrasound
EMR	endoscopic mucosal resection
ESD	endoscopic submucosal dissection
CSP	cold snare polypectomy
PPI	proton pump inhibitor
NSAIDs	nonsteroidal anti-inflammatory drugs
FCSEMS	fully covered self-expanding metal stents
